# The effects of Digital Buddy programme on older adults’ mental well-being: study protocol for a multi-centre, cluster randomized controlled trial

**DOI:** 10.1186/s13063-023-07130-5

**Published:** 2023-02-07

**Authors:** Rick Yiu Cho Kwan, Fowie Ng, Manfred Lai, David Wong, Sally Chan

**Affiliations:** 1 School of Nursing, Tung Wah College, Hong Kong SAR, China; 2 School of Management, Tung Wah College, Hong Kong SAR, China; 3 President’s Office, Tung Wah College, Hong Kong SAR, China; 4 JC School of Public Health and Primary Care, The Chinese University of Hong Kong, Hong Kong SAR, China

## Abstract

**Introduction:**

Mental well-being is associated with many mental health symptoms, including depression and health-related quality of life. Digital divide could impact mental health, particularly during the COVID-19 pandemic. Information and communication technology (ICT)-based tools and interventions could effectively provide social support. Intergenerational mentoring between college students and older adults could promote eHealth literacy and self-efficacy, and it is advocated to bridge the digital divide for older adults. However, the effectiveness of an intervention which employs ICT-based tools and intergenerational mentoring strategies (i.e. Digital Buddy) on mental well-being is unclear.

**Methods:**

This study will employ a multi-centre, cluster-randomized, two-parallel-group, noninferiority, controlled trial design with a 1:1 group allocation ratio. In the intervention group, a Digital Buddy (i.e. a young volunteer) is assigned to a group of older adults in a 1:10 ratio. A series of training sessions for a minimum of 23 h will be provided to the older adults by Digital Buddy, who will also follow through the intervention period with the older participants. The training contents include ICT and mental health care knowledge and skills. The whole intervention period will last for 6 months between 14 sessions. In the control group, participants will receive the usual care. The primary outcome measure is mental well-being. We aim to recruit 292 older participants. Generalized estimating equations (GEE) will be used to examine the effects of the intervention.

**Ethics and dissemination:**

This trial has been registered at ClinicalTrials.gov (NCT05553730) on 23 September 2022, https://clinicaltrials.gov/ct2/show/NCT05553730, and all items come from the World Health Organization Trial Registration Data Set. It has been approved by the Research Ethics Committee of Tung Wah College, Hong Kong (reference number: REC2022143). The findings will be disseminated in peer-reviewed journals and presented at international conferences relevant to the subject fields.

## Introduction

Mental well-being, as defined by WHO, is a state where people realize their abilities, can cope with the normal stress of life, can work productively, and can contribute to their community [1]. It is more than the absence of mental disorders or disabilities. The stress of life across the lifespan (e.g. the COVID-19 pandemic, organizational stress) is known to be associated with poor mental well-being [2, 3]. Mental well-being is associated with many health mental health symptoms, including depression and health-related quality of life [4, 5]. Under the influence of the prolonged COVID-19 pandemic and its related policies (e.g. quarantine, lockdown), older adults’ mental well-being is negatively affected [6]. A recent study in Hong Kong showed that older adults experienced a much higher level of depressive symptoms compared with the pre-pandemic period [7, 8].

Digital divide refers to inequalities of access to information and communication technologies (ICT), such as the ability to use computers, smartphones, or the internet, because of demographic differences (e.g. age, income, education) [9]. During the pandemic period, even in the developed world, difficulties in the utilization of digital healthcare among older adults continued to be observed [10]. Latest surveys in Hong Kong found that smartphone ownership and internet usage among those aged 65 or above have increased from 57.2% and 56.3% in 2018 to 68.1% and 65.9% by the second half of 2020. Yet, these figures are still far below those of younger age groups, which have capped at > 98% [11]. A study explicitly showed that the digital divide between older adults (age > 75 years) and their counterparts exist in terms of internet use for health information (OR = 0.37) [12]. Albeit the fact that mental well-being is known to be associated with classic health behaviours, such as physical activity and body-mind exercise [8, 13]; recent studies suggested that the digital divide could impact mental health, particularly during the COVID-19 pandemic [14].

Social support refers to the social resources available in the context of formal support groups and informal helping relationships [15]. Evidence suggests that social support is positively associated with mental well-being, particularly in vulnerable people [16, 17]. A longitudinal study showed that the mental well-being of older adults improved significantly after the gradual recovery of social support services during the relenting COVID-19 pandemic in the UK [18]. A systematic review showed that ICTs-based tools and interventions could effectively provide social support [19]. Therefore, enhancing social support through the provision of ICT-based tools and interventions could promote mental well-being.

Self-efficacy refers to an individual’s belief in their capacity that they can influence the results [20]. Evidence shows that self-efficacy is positively associated with mental well-being [21]. A study showed that a training programme is effective to enhance older adults’ self-efficacy and attitudes toward computers and the internet [22]. With well-planned instructions, the uptake and feasibility of ICT-based interventions are very high [23–25].

Intergenerational mentoring refers to the positive aspects of reverse mentoring without the hierarchical framework of mentor and mentee in that it focuses on the belief that everyone leads and everyone learns [26]. Intergenerational mentoring between college students and older adults could promote eHealth literacy and self-efficacy, and it is advocated to bridge the digital divide for older adults [27]. Evidence suggests that older adults could acquire new digital skills through explicit and on-explicit learning dynamics in their interaction with the young people in their families [28].

To summarize the evidence in the literature illustrated above, the digital divide in older adults may potentiate the detrimental effects of the COVID-19 pandemic on mental well-being and eventually on their health-related quality of life. A programme of intergenerational mentoring coupled with ICT training aiming at bridging the digital divide, providing better social support, and promoting self-efficacy may be a solution to promote the mental well-being and health-related quality of life of older adults. However, evidence of these hypothetical relationships is lacking in the literature. Therefore, this study aims to evaluate the effectiveness of a Digital Buddy programme on the mental well-being, depressive symptoms, health-related quality of life, self-efficacy, and social support of older adults. This study will yield knowledge about the effects of a new intervention modality to promote the mental well-being of older adults in places with the digital divide.

## Methods

This randomized controlled trial protocol reports the methods following the Standard Protocol Items: Recommendations for Interventional Trial (SPIRIT) 2013 guideline [29].

### Trial design

This study will employ a multi-centre, cluster-randomized, two-parallel-group, noninferiority, controlled trial design with a 1:1 group allocation ratio.

### Study setting

The study will be conducted in facilities for older adults, including community centres and/or long-term care facilities for older adults in Hong Kong. Community centres for older adults provide various activities aiming to enable older adults to remain in the community and to lead a healthy, respectful, and dignified life [30]. Long-term care facilities for older adults provide residential care, meals, personal care, and nursing care for older adults who suffer from poor health or physical/mild mental disability [31].

### Participants

A convenience sampling approach will be employed. The eligibility criteria of the participants are below:

#### Eligibility criteria

1. Aged 60 years or above,
2. Speak Cantonese and read traditional Chinese characters, and
3. Mentally capacitated is defined as having no diagnosed mental diseases leading to being mentally disabled and certified by a psychiatrist

### Interventions

#### Intervention group

The goal of the intervention is to enhance the ICT skills and promote the mental well-being of the older participants. A series of training sessions for a minimum of 23 h will be provided to the older adults. In the training sessions, the Digital Buddy will lead older adults to complete activity-based ICT tasks on their smartphones and answer questions they may have about smartphone usage. In the second half of the session, Digital Buddy will review the mental health contents assigned for each week and bring up group processing questions to encourage older adults to reflect on their attitudes about well-being and ageing. As shown in Table 1, the scope of the ICT skills includes foundational training for smartphone operation, usage of common social media platforms and safe web navigation for accessing information, while the scope of the mental health promotion includes stress relief, breathing and well-being, emotion regulations, and positive ageing. The whole intervention period will last for 6 months between 14 sessions. A systematic review of intergenerational interactions showed that the intervention programme length ranged from three months to three years but a six-month period is more commonly used for student-led programmes because it is more manageable for students within one school year [32].

**Table 1:** ICT skills and mental health contents in the training sessions

Session	Duration	Training contents	
		ICT skills	Mental health
1	2 h	Introductory lesson	
2	2 h	1. Use of screen and operation buttons 2. Add phone contact 3. Review missed calls 4. Connect to internet	1. Review common symptoms in ageing adults 2. Practice a sensory relaxation technique 3. Engage in group processing questions
3	2 h	1. Use of camera functions 2. Use of a selfie camera 3. Create and organize photo album 4. Share photos on social media apps	1. Review tips to prevent anxiety 2. Practice the CABIN Relaxation Technique 3. Engage in group processing questions
4	2 h	1. Operate timer 2. Use of calculator 3. Use of voice-activated keyboard 4. Learn new functions of the LeaveHomeSafe App	1. Practice a 5-min guided mediation 2. Engage in group processing questions
5	1.5 h	1. Operate WhatsApp 2. Type and voice message 3. Share photo 4. Forward/delete message	1. Practice light stretching 2. Engage in group processing question
6	1.5 h	1. Register account 2. Compose an email 3. File attachment 4. Junk box	1. Practice pain relief massage 2. Engage in group processing question
7	1.5 h	1. The search function on YouTube 2. Like and favourite function 3. Create playlist	1. Practice abdominal breathing 2. Engage in group processing question
8	1.5 h	1. Create Zoom account 2. On and off for microphone and video camera 3. Write messages in a chat room	1. Practice hand-eye coordination exercise 2. Engage in group processing question
9	1.5 h	1. Download and operate HA GO app 2. Register an account 3. Book an appointment	1. Practice light cardio exercise 2. Engage in group processing question
10	1.5 h	1. Functions of the HealthCap app 2. Introduction to e123.hk	1. Practice breathing exercise 2. Engage in group processing question
11	1.5 h	1. Introduction to elderly.gov.hk 2. Introduction to elderlyinfo.swd.gov.hk	1. Practice a guided cognitive exercise 2. Engage in group processing question
12	1.5 h	1. Set and reset the password on the smartphone 2. Strategies to use the web safely	1. Practice posture-improving exercise 2. Engage in group processing question
13	1.5 h	1. Use of Samsung Health app 2. Practice light stretching exercises	1. Practice upper body stretching exercise 2. Engage in group processing question
14	1.5 h	Conclusion remarks	
Total	23 h		

The intervention will match up the older adults with the young adults (i.e. the Digital Buddy) in a ratio of 1 (young adults) to 10 (older adults). The study will recruit Digital Buddy if they are aged between 18 and 29 years and studying a full-time programme in a tertiary institution. The Digital Buddy will provide the training in a flexible group size, at a flexible place, and at a flexible time aiming to accommodate the availability of both older and young adults. A train-the-trainer programme will be provided by the research team to the young adult participants. The young adult participants will be qualified to become Digital Buddies in this programme after they have completed a 2-h train-the-trainer programme. The contents of the train-the-trainer cover the activities used to promote ICT skills and mental health promotion knowledge, as well as the essential communication skills with older adults.

**Fig. 1:**
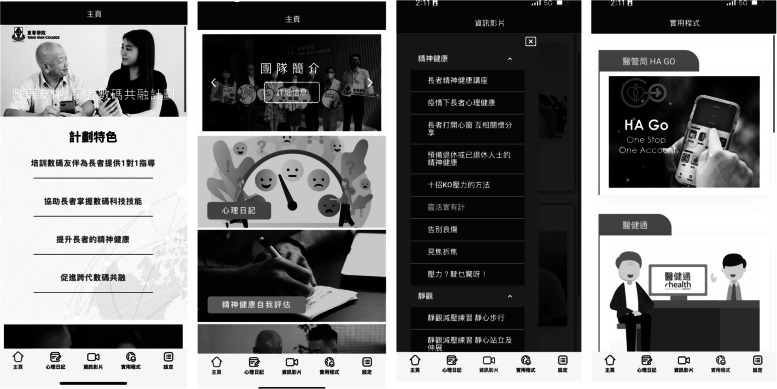
Training materials uploaded to a newly developed web-based platform

The training materials are uploaded to a newly developed web-based platform (www.twcdigitalbuddy.hk), as shown in Fig. 1. This web-based platform contains all the educational materials in Chinese related to ICT skills and mental promotion in various forms (e.g. video-based mini-lectures, micro-movies, and video-guided demonstrations). The educational materials are either newly produced by the team or already available on the web, yet they are all reviewed and content-validated by an expert panel. After equipping the older participants with the ICT skills so that they can independently exercise these skills to communicate with the Digital Buddy, the Digital Buddy will deliver the training sessions or necessary coaching in person with the option to communicate on e-platforms (e.g. WhatsApp, Zoom). Both the older participants and the Digital Buddy will access the app-based training materials, attend coaching sessions, and communicate using smartphones over the course of the six months programme.

To ensure satisfactory adherence to the intervention of the self, the project team will telephone contact both the Digital Buddy and the older participants at least once weekly to identify their difficulties participating in the intervention and provide administrative support to help resolve their problems. If some participants within the same cluster miss some training sessions, they will be invited to attend maximally two make-up training sessions after the completion of the 23-h scheduled training programme. In the make-up session, the training contents will be tailor-made for what they have missed. To ensure the availability of access to the Internet, older participants who do not own a smartphone will be provided with a smartphone with a data package complementarily during the study period. There will be no special criteria for discontinuing or modifying allocated interventions.

#### Control group

Participants in the group will receive the usual care. The research team will not provide any form of support to the participants in this group.

### Outcomes

Demographic data will be collected at baseline (T0), including age, gender, education attainment, employment status, financial satisfaction, living arrangement, years of smartphone usage, daily average of internet usage, perceived ability to use a smartphone, and perceived ability to use the internet. Five outcome markers will be measured at both baseline (T0) and the week immediately after the completion of the intervention (T1). The primary outcome is mental well-being. Four secondary outcomes will also be measured, including depressive symptoms, health-related quality of life, self-efficacy, and social support.

#### Mental well-being (primary outcome)

The World Health Organization Five Well-being Index (WHO-5) will be used to measure mental well-being over the past two weeks [33]. WHO-5 comprises five items and each item is rated by a 6-point Likert scale from 0 (at no time) to 5 (all of the time). The total score ranges from 0 to 25. A higher score indicates an increased sense of psychological well-being. The Cantonese version of WHO-5 showed good internal consistency (*α* = 0.86) and good concurrent validity with quality of life (*r* = 0.41–0.51) [33].

#### Depressive symptoms

The 9-item Patient Health Questionnaire (PHQ-9) will be used to measure depressive symptoms over the past two weeks [34]. PHQ-9 comprises nine items and each item is rated by a 4-point Likert scale from 0 (not at all) to 3 (nearly every day). The total score ranges from 0 to 27. A higher score indicates greater severity of depressive symptoms. The Cantonese version of PHQ-9 showed good internal consistency (*α* = 0.82), test-retest reliability (*r* = 0.76), and satisfactory concurrent validity with the mental component of quality of life (*r* = − 0.60) [34].

#### Health-related quality of life

The 12-item Short Form Health Survey Version 2 (SF-12v2) will be used to measure health-related quality of life over the past four weeks [35]. SF-12v2 comprises 12 items rated by scales with varying points measuring eight domains of health, including physical functioning, role physical, bodily pain, general health, vitality, social functioning, role emotional, and mental health. The scores of eight domains are aggregated into two scores: (1) physical component summary score and (2) mental component summary score. The scores will be calculated by a standard algorithm [36]. A higher component summary score indicates a better health-related quality of life. The Chinese version of SF-12v2 showed good test-retest reliability (*r* = 0.67–0.82), and good construct validity with >80% of total variance explained by the two-factor model [37, 38].

#### Self-efficacy

The 10-item General Self-Efficacy Scale (GSES-10) will be used to measure self-efficacy and it was translated and adapted to Chinese. GSES-10 comprises 10 items and each item is rated by a 4-point Likert scale from 1 (not at all true) to 4 (exactly true). The total score ranges from 10 to 40. A higher score indicates better self-efficacy. The Chinese version GSES-10 showed good internal consistency (*α* = 0.91) [39], good criterion validity with wellbeing (*r* = 0.56–0.63, *p* < 0.001), self-esteem (*r* = 0.40–0.49, *p* < 0.001), and mental health (0.39–0.41, *p* < 0.001) [40].

#### Social support

The 12-item Multidimensional Scale of Perceived Social Support (MSPSS-12) will be used to measure perceived social support [41]. MSPSS-12 comprises 12 items which are factored in three subscales by sources of support (i.e. family, friends, significant others). Each subscale comprises four items. Each item is rated on a 7-point Likert scale from 1 (strongly disagree) to 7 (strongly agree). The total score ranges from 12 to 84. A higher score indicates better social support. The Chinese version of MSPSS-12 showed good internal consistency (Cronbach’s *α* = 0.95), good test-retest reliability (ICC = 0.91), and satisfactory concurrent validity with perceived stress (*r* = − 0.221) and caregiving rewarding feelings (*r* = − 0.327) [42].

### Participant timeline

As shown in Table 2, participant recruitment and consenting will take place in the 4 weeks before the baseline. Data collection of demographic and outcome data will take place at the baseline (T0). Interventions including the Digital Buddy and usual care will take place in the intervention period in weeks 1–24. Outcome data will be collected in week 25. Participants allocated to the usual care group will be given a waitlist intervention in weeks 26–49.

**Table 2:** Schedule of enrolment, intervention, and assessments (SPIRIT figure)

	Study period			
	Enrolment	Allocation	Post-allocation	Close-out
Week	− 4	0	1–24	25
Timepoint	T0			T1
Enrolment				
Eligibility screen	x			
Informed consent	x			
Allocation	x			
Interventions				
Digital Buddy			x	
Usual care			x	
Assessment				
Demographic	x			
Mental well-being	x			x
Depressive symptoms	x			x
Health-related quality of life	x			x
Self-efficacy	x			x
Social support	x			x

### Sample size

We adopt a prior power analysis using the GPower employing the statistical test of the mean difference between two different groups. We set the level of significance at 0.05, the power at 0.8, and the allocation ratio between the two groups at 1:1. To estimate the effects, we refer to a similar pilot trial examining the effect of an intergenerational mentoring programme [43]. The effect size (i.e. Cohen’s *d*) of the study of the intervention on the primary outcome (i.e. well-being) was 0.5. A sample size of 102 participants is needed. We adopt Hemming’s method to adjust the sample size required under individual randomization for cluster RCT with fixed and equal-sized number clusters [44]. Considering that we will collect data in 6 clusters and assuming that the intra-cluster correlation coefficient (ICC) is 0.01, the estimated total sample size is 244 (i.e. 122 per arm). We assume that the drop-out rate is approximately 20%. A total sample of 292 is estimated, with 146 participants in each arm.

### Recruitment

Promotional seminars and booths will be conducted in the facilities for older adults (e.g. community centres for older adults, residential care homes for older adults) and tertiary institutions. In the seminars, participants will be directly recruited. The programme will be introduced to collaborative organizations. Both printed and electronic posters will be produced and sent to the members of the collaborative organizations. The staff members of the collaborative organizations will help recruit their members to participate in this project. The estimated rate of recruitment is 90% because most of the participants could fulfil the eligibility criteria and they self-enrol to participate after reading the information that they are highly likely to give consent to participate in the study.

### Assignment of interventions

**Fig. 2:**
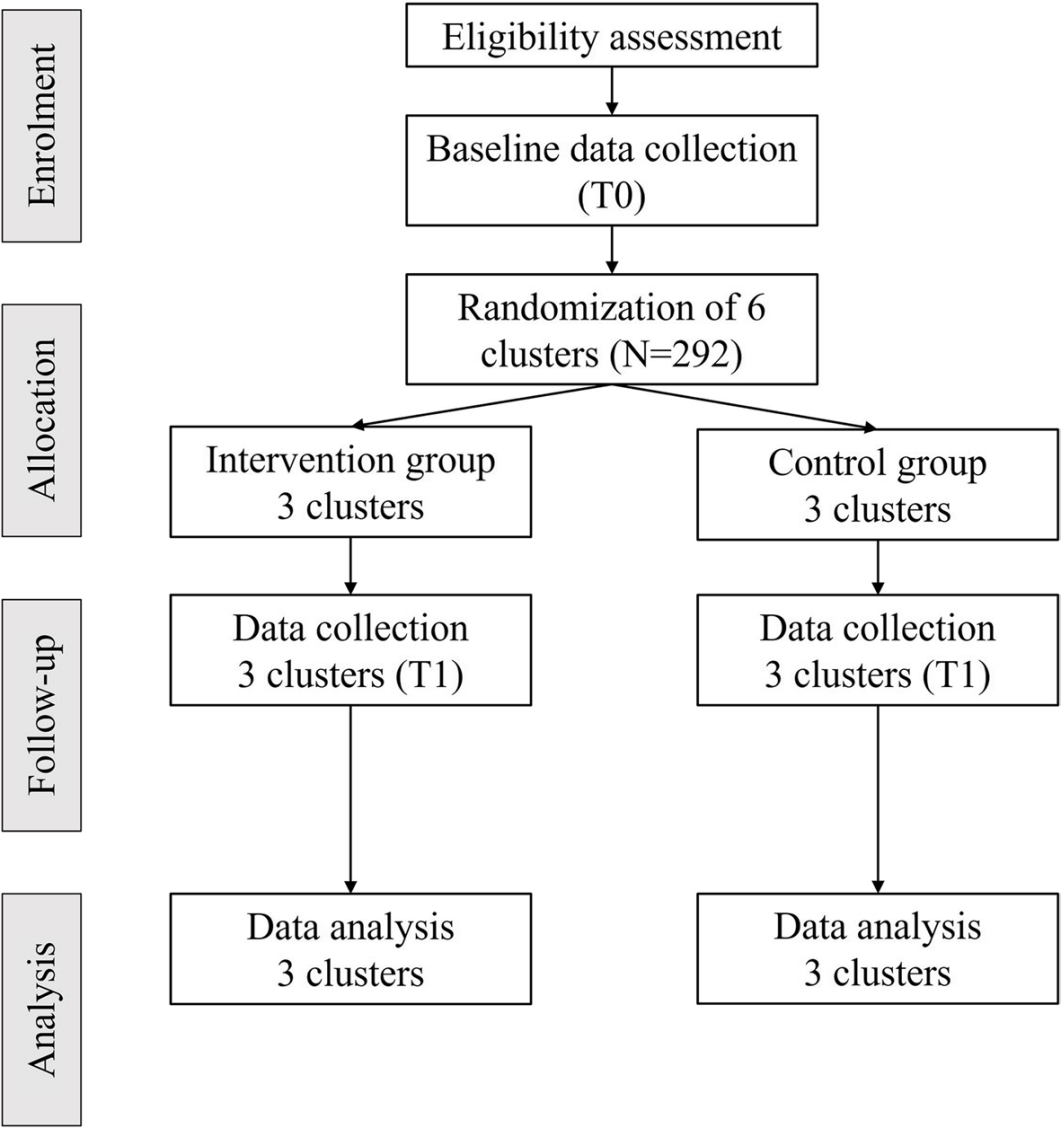
Cluster randomization method

As shown in Fig. 2, the cluster randomization method was employed to reduce the risk of within-cluster contamination because most of the training materials are open source that participants share with their peers in a cluster [45]. A permuted block (block size = 6) random allocation sequence list with an allocation ratio to either intervention group or control at 1:1 will be generated by the web-based application www.random.org. The cluster unit of randomization is based on a facility for older adults (e.g. one community centre as a unit). Each unit will be randomly assigned after the entire unit is screened, written consent is obtained, and baseline assessment is completed to reduce the risk of selection bias [46]. A group of six units (i.e. the size of a permuted block) with a similar number of eligible and consented participants will be grouped to be randomly allocated. This aims to ensure each group comprises a similar number of participants. The random allocation process will be implemented by an independent statistician, who will not participate in any other parts of the proposed study. The statistician will assign group labels to each unit based on their sequence of entries with reference to the sequence list, thus ensuring that other members of the research team cannot foresee the group allocations. In this study, only the outcome assessor will be blinded to the group labels. It will not be possible to blind the participants or the interventionists. In the intervention group, we will conduct interventions for the three clusters simultaneously when we have secured the study venues and done the randomization.

A waitlist control design will be adopted so that the participants randomized into the control group will be provided with a chance to receive the intervention after they have completed their untreated condition (i.e. receiving usual care for 6 months).

### Statistical methods

Demographic and outcome data at baseline will be described either as means with standard deviation or as frequencies with percentages based on the level of measurement. Five generalized estimating equations (GEE) will be used to separately test the five outcomes. To test the hypotheses, the dependent variables will be mental well-being, depressive symptoms, health-related quality of life, self-efficacy, and social support across the two time points. The independent variables of the four hypotheses will be the same: group (i.e. intervention and control groups), time (i.e. T0 and T1), and group × time. The interpretation of the results will be based on intention-to-treat analysis [47]. We will primarily interpret the results using models without adjusting for possible co-variates because this will be an RCT without other known or anticipated important prognostic variables [48]. The level of significance will be set at 0.05. To manage missing data caused by unavoidable reasons (e.g. drop-out), the missing data will be replaced by multiple imputations following Jakobsen’s algorithm [49]. We will adopt an intention-to-treat analysis to conclude the intervention effects [47].

### Oversight and monitoring

A trial steering team of three academics in the disciplines of nursing and occupational therapy (i.e. the authors RK, FN, SC) will direct the whole study process. The project implementation team comprising one project officer and one project assistant will be responsible to provide day-to-day support for the trial. An advisory panel comprising one geriatrician and one social worker specializing in gerontology will provide advice to the project. The trial steering team will meet the project implementation team bi-weekly to monitor the quality of implementation. The advisory panel, which is also the data monitoring committee, comprises one geriatrician and one academic specialized in gerontological social work, who are independent of the sponsor and competing interests, the trial steering team, and the project implementation team. The trial steering team reports to the advisory panel, which will meet the trial steering team once per three months to advise on upholding the quality of the trial (e.g. data collection and analysis quality). If actual or potential harms are identified, the panel will decide to consider suspending or terminating the trial.

### Data collection and management

Data will be collected by the project implementation team after they have completed the training provided by the trial steering team. To prevent data missing, a computerized data collection platform (i.e. www.qualtrics.com) will be used, and the system will prompt the data collectors for unfilled data fields or the data collectors must provide reasons for leaving the data fields empty. Immediately after the data collection, preliminary data analysis will be conducted by the research team to do the range checks for data values. When out-of-range data are observed, the research team will investigate the reasons and implement rectifications (e.g. re-do the data collection whenever possible). Qualtrics employed many methods for the protection of customer data (e.g. high-end firewall system, Transport Layer Security encryption [50]. The Qualtrics data are also password-protected. After each round of data collection, the data on Qualtrics will be downloaded and saved on the password-protected cloud server at Tung Wah College for at least 7 years according to the requirement of the ethics committee. After the completion of the study, only the research team members (i.e. RK, FN, SC) have right to access the final dataset.

### Ethics and dissemination

This trial has been registered at ClinicalTrials.gov (NCT05553730) on 23 September 2022, https://clinicaltrials.gov/ct2/show/NCT05553730, and all items come from the World Health Organization Trial Registration Data Set. There is no anticipated harm or compensation for trial participation. The participant information materials and informed consent form are available from the corresponding author on request. The findings will be disseminated in peer-reviewed journals and presented at international conferences relevant to the subject fields. In case there is a protocol amendment, the research team will notify the sponsor and funder first. Then, the centres will be notified with a copy of the revised protocol. If there are any deviations from the protocol, they will be fully documented using a breach report form and the details in the clinical trial registry will also be updated.

## Discussion

Mental well-being is an important health outcome in older adults as stated in the Comprehensive Mental Health Action Plan 2013-2030 [51]. Evidence showed that activity-based interventions (e.g. Yoga, community singing) are effective to promote the well-being of older adults [13, 52]. However, the acceptability of these activities is interest-selective in that they may not be accepted or up-taken prolongedly by older adults across cultures. Preventive lifestyle interventions are more universally adoptable, but a study showed that a preventative lifestyle intervention (e.g. discussion, activities, and community enactment) was ineffective in promoting the mental well-being of older people [53]. This intervention aiming at promoting older people’s mental well-being through new components (i.e. promoting ICT skills and intergeneration mentorship) is theoretically effective but empirically robust. This trial will generate new knowledge on the effects of this robust intervention on promoting mental well-being.

Most of the Digital Buddies will be first-year undergraduate students and likely to have little to no experience volunteering with older adults. They may find it difficult to encourage older adults to participate in activities and lead group-sharing discussions where each participant has the chance to participate. To ensure Digital Buddies and older adult participants are well supported, at least one project staff member will be present at the training sessions to help clear up uncertainties or difficult requests. Project staff will observe session progress and report participant feedback to the trial steering team on a bi-weekly basis.

A volunteer guideline and FAQ were developed to bring clarity about the role and responsibilities of Digital Buddies. The documents seek to help Digital Buddies understand the boundaries they should maintain as youth volunteers and offer strategies to deal with difficult situations they may encounter when volunteering with older adults.

## Trial status

Recruitment is expected to begin on the 25 November 2022 and until 28 April 2023.

## Data Availability

The data will be available upon request.

## Abbreviations

WHO: World Health Organization
COVID-19: Coronavirus disease of 2019
ICT: Information and communication technology
WHO-5: World Health Organization Five Well-being Index
PHQ-9: 9-Item Patient Health Questionnaire
SF-12v2: The 12-item Short Form Health Survey Version 2
GSES-10: 10-item General Self-Efficacy Scale
MSPSS-12: 12-item Multidimensional Scale of Perceived Social Support
